# Notes for July Journal

**Published:** 1870-07

**Authors:** 


### NOTES FOR JULY JOURNAL.

"Drinking Fountain Stories," published by the National Temperance Society and Publication House, 172 William St., New York.

"Tabulated Mortuary Record of Savannah, Georgia, from January 1, 1854, to December 31, 1869," by Wm. Duncan, M. D.

"The first Annual Report of the American Museum of Natural History for 1780." John David Wolfe, President. A. J. Phelps Dodge, Secretary. This Museum will be located in the Central Park. $45,000 have been already expended through the judicious agency of William T. Blodgett. Coleman T. Robinson has presented to the Museum a collection of Lepidoptera, consisting of about 3,000 species, represented by at least 10,000 specimens, with a valuable library of books connected with natural history. The enterprise was inaugurated and has been liberally sustained by our best, wealthiest, and most enterprising citizens; among these are James Brown, of the firm of Brown Brothers, John

David Wolfe, A. T. Stewart, Arnold, Constable & Co., who are the largest of the contributors by double.

The American Tract Society, 150 Nassau St., New York, has issued some works of great interest to the young; one or two of these will be sent for ten cents by mail, post paid, by addressing as above. Among these are "A Sound Mind," "Man and his Masters," by John B. Gough, "Law of Labor and Love," "Young Men beginning Life," "Life a Race," "Dignity of Labor," "Defaulters," "The Mother's Sorrow," by Rev. Charles Wadsworth, D. D., "The Young Men Succeeding or Failing in Business," "The Young undecided in Religion," "Independence of Mind," "The Imagination, its Uses and Abuses," by Rev. James McCosh, LL. D.

"The New Englander," published at $4 a year, issued quarterly at New Haven, Conn. Edited by Professors Fisher and Dwight, of Yale College, and Rev. William L. Kingsley.

"The Technologist," especially devoted to engineering, manfacturing and building, issued monthly by the Industrial Publication Company, 176 Broadway, New York, at $2 a year. The May number presents a rich table of contents, with several fine engravings, and is well worthy of a large circulation, its mechanical getting up is creditable to the company. It is certainly a very instructive publication.

"The Manufacturer and Builder," $1.50 a year, issued monthly, 15 cts. each, by Western & Co., 37 Park Row, is another publication for the industrial classes, tending to elevate mechanics and mechanical science by the diffusion of all knowledge, among working men, calculated to fit them for their calling, so that they may work intelligently, and not in that tread-mill, stereotype manner, which is the bane of improvement. Every mechanic owes it to himself to take one or more publications of this kind as a means of improvement, elevation and progress in his calling, however humble that may be.

"The American Builder," $3 a year, published at Chicago, 115 Madison St., by Charles D. Lakey, is another monthly, and commends itself to the patronage of Architects, Contractors, Carpenters and Masons, wherever they reside.

"London Lancet," $5 a year, republished by Wm. C. Hazzard, 52 John St., New York, devoted to medical and surgical progress throughout the world.

"A simple, uniform, Decimal System of Weights and Measures and Coinage." Address H. Edgar Johnson, 35 North Charles St., Baltimore, Maryland, United States of America. The casual reader may be inclined to pass this over as of no possible interest to himself. But it is a subject which ought to interest every intelligent mind. To have the same weights, measures, and money, called by the same names all over the world, and counted by tens, would save an incalcuable amount of brain bother, and of time in making calculations. It will be a benevolence, a charity towards human kind, for the reader to send ten, fifty, a hundred or a thousand dollars, to the address given, to aid in publishing information and putting forth the means necessary to secure the success of the measure. Half the work is already done.

